# A Comparison of the Tibial Intraosseous Route With the Peripheral Intravenous Route for Fluid Resuscitation of Patients in Hypovolemic Shock

**DOI:** 10.7759/cureus.91438

**Published:** 2025-09-01

**Authors:** Mahima Agarwal, Nikki Sabharwal, Arin Choudhury, Naresh K Gautam

**Affiliations:** 1 Anaesthesia and Intensive Care, Vardhman Mahavir Medical College and Safdarjung Hospital, New Delhi, IND

**Keywords:** hypovolemic shock, intraosseous, peripheral intravenous, resuscitation, vascular access

## Abstract

Aim and objectives

Vascular access via the intraosseous (IO) route is increasingly utilized in patients with hypovolemic shock, especially when peripheral intravenous (IV) access is challenging due to collapsed veins. The present study aims to compare the tibial intraosseous route with the peripheral intravenous route for fluid resuscitation in adults with hypovolemic shock (Class >=II) in an emergency setting. The primary objective of this study is to compare the first attempt success rate and time taken to establish initial vascular access via the IO and IV routes. The secondary objective of this study is to compare the ease of establishment of the two routes using a five-point Likert scale.

Material and methods

The study included adult patients (18-65 years old) in hypovolemic shock (Class >=II) presenting to the emergency department, who required immediate vascular access. The patients were randomized to one of the two routes of vascular access: tibial intraosseous or peripheral intravenous. Outcome variables included the first attempt success rate, time taken to establish vascular access, and the ease of establishing the IO route (using the EZ-IO^®^ device (Teleflex, Morrisville, NC) as compared to the IV route.

Results

Out of 70 patients, 35 (50%, Group I) received proximal tibial vascular access via EZ-IO device, and 35 (50%, Group II) received peripheral intravenous access. First attempt success rate was significantly higher in Group I (100%) than in Group II (45.71%, (p<0.0001).

The median time taken to establish vascular access (in seconds) was 77 (76-80) and 83 (49-99) in Groups I and II, respectively (p=0.830). This difference was statistically insignificant but clinically significant. The IO route was significantly easier to establish than the IV route as per a five-point Likert scale (p<0.0001).

Conclusion

Tibial intraosseous access has a higher first attempt success rate, is faster, and easier to establish than the peripheral intravenous route.

## Introduction

The establishment of vascular access is vital for life-saving interventions in patients with severe trauma or critical illness. In cases of hemorrhagic shock, delays in administering fluids and blood products can significantly worsen patient outcomes. However, achieving intravenous (IV) access in emergencies can be challenging, as shock often leads to the collapse of peripheral blood vessels. In such situations, intraosseous (IO) access presents a valuable alternative by delivering fluids directly into the non-collapsible venous plexus of the medullary cavity at flow rates that are comparable to the IV access, when a pressure bag is used [1-7].

The IO route of vascular access was first proposed by Drinker et al. in 1922, but its use had been primarily limited to pediatric resuscitation [8]. However, by the late 1990s, the introduction of new mechanical IO devices led to a broader adoption of IO access [9]. Recently, several guidelines, including those from Advanced Trauma Life Support (ATLS), Advanced Cardiac Life Support (ACLS), the European Resuscitation Council, and the Indian Resuscitation Council Federation (IRCF)-have recommended IO access when IV routes are difficult to establish. IO access has shown particular effectiveness in prehospital and combat scenarios, where IV insertion is less feasible [10,11]. 

The most commonly used site for IO access is the proximal tibia. Other less commonly used sites include the distal tibia, proximal humerus, sternum, distal femur, spine, calcaneum, and radius [1,2,4,7,12]. Contraindications to IO access include a fractured target bone, infection or burn at the insertion site, prosthetic limb, recent surgical procedure in the target limb, metabolic bone diseases (e.g., osteogenesis imperfecta, osteoporosis), and previous IO access attempt in the target bone within the past 48 hours [1,3,7]. Though rare, complications of IO access can include extravasation of fluids (leading to compartment syndrome), osteomyelitis, cellulitis, localized abscess formation, growth plate abnormalities, air/fat embolism, and myonecrosis with hypertonic saline infusion [2-5,13,14]. It is recommended to keep the IO line in place for a maximum of 24 hours, replacing it with a peripheral intravenous line as soon as possible to avoid long-term complications [2].

Despite the growing acceptance of IO access in emergency and trauma care, research comparing the outcomes of IO versus IV access, particularly in patients with severe trauma experiencing traumatic cardiac arrest or hemodynamic instability, remains inconclusive [15-17]. While some studies highlight the success of IO access, the results are mixed and the available research is still insufficient. Although the use of IO injections has grown globally, they are likely still underused. In India, the trauma care system has made significant advancements over the past decade, contributing to a reduction in preventable trauma-related deaths. However, there is limited research on the frequency and adoption of IO access in emergency settings.

This study aims to evaluate the tibial IO route compared to peripheral IV access in adult patients presenting with hypovolemic shock (class >=II) in the emergency department. The focus is on comparing the first-attempt success rate, time taken to establish vascular access, and ease of fluid resuscitation. The objective is to determine whether IO access offers a superior alternative to peripheral IV access in these critically ill patients.

## Materials and methods

Study design and setting

This was a prospective, interventional, randomized study conducted in the emergency department of the hospital by trained physicians over a period of 18 months from December 2020 to May 2022, after approval from the institutional review board and ethics committee. Written informed consent was obtained from the patient/patient’s relative(s) before the intervention was performed.

Selection of participants

The study included patients aged 18-65 years of either gender, weighing >40 kg who presented to the emergency department with hypovolemic shock of class >=II (both traumatic and non-traumatic causes), defined by fluid loss exceeding 15% of total blood volume, heart rate greater than 100 BPM, and respiratory rate over 20/min. The ATLS classification of hypovolemic shock is shown in Table 1.

**Table 1:** ATLS classification of hypovolemic shock ATLS: Advanced Trauma Life Support.

	CLASS I	CLASS II	CLASS III	CLASS IV
Blood loss in %	<15	15-30	30-40	>40
Pulse Rate (BPM)	<100	100-120	120-140	>140
Blood Pressure	Normal	Normal	Normal	Decreased
Pulse Pressure	Normal or Increased	Decreased	Decreased	Decreased
Respiratory Rate	14-20	20-30	30-40	>35
Mental Status	Slightly Anxious	Mildly Anxious	Anxious, Confused	Confused, Lethargic
Urine Output (mL/hr)	>30	20-30	5-15	Minimal

Patients excluded from the study were those with already established peripheral vascular access or a previous attempt at IO access in the target bone within the past 48 hours, those with infection/ burn/ fractured target bone/ prosthetic limb in the target area, patients who had recently undergone surgery on the target limb, those with metabolic bone diseases such as osteogenesis or osteoporosis and patients experiencing shock due to causes other than hypovolemia.

Interventions

Reades et al. conducted a randomized trial involving 182 adult patients to compare the first-attempt success rates of the tibial IO route with the peripheral intravenous route. The first attempt success rate for tibial vascular access was 91% and that of the peripheral intravenous route was 43% [12]. Taking this study as a reference, the required sample, taking power of the study as 80% and a 5% level of significance, was 35 in each study group (Total sample size=70).

**Figure 1:**
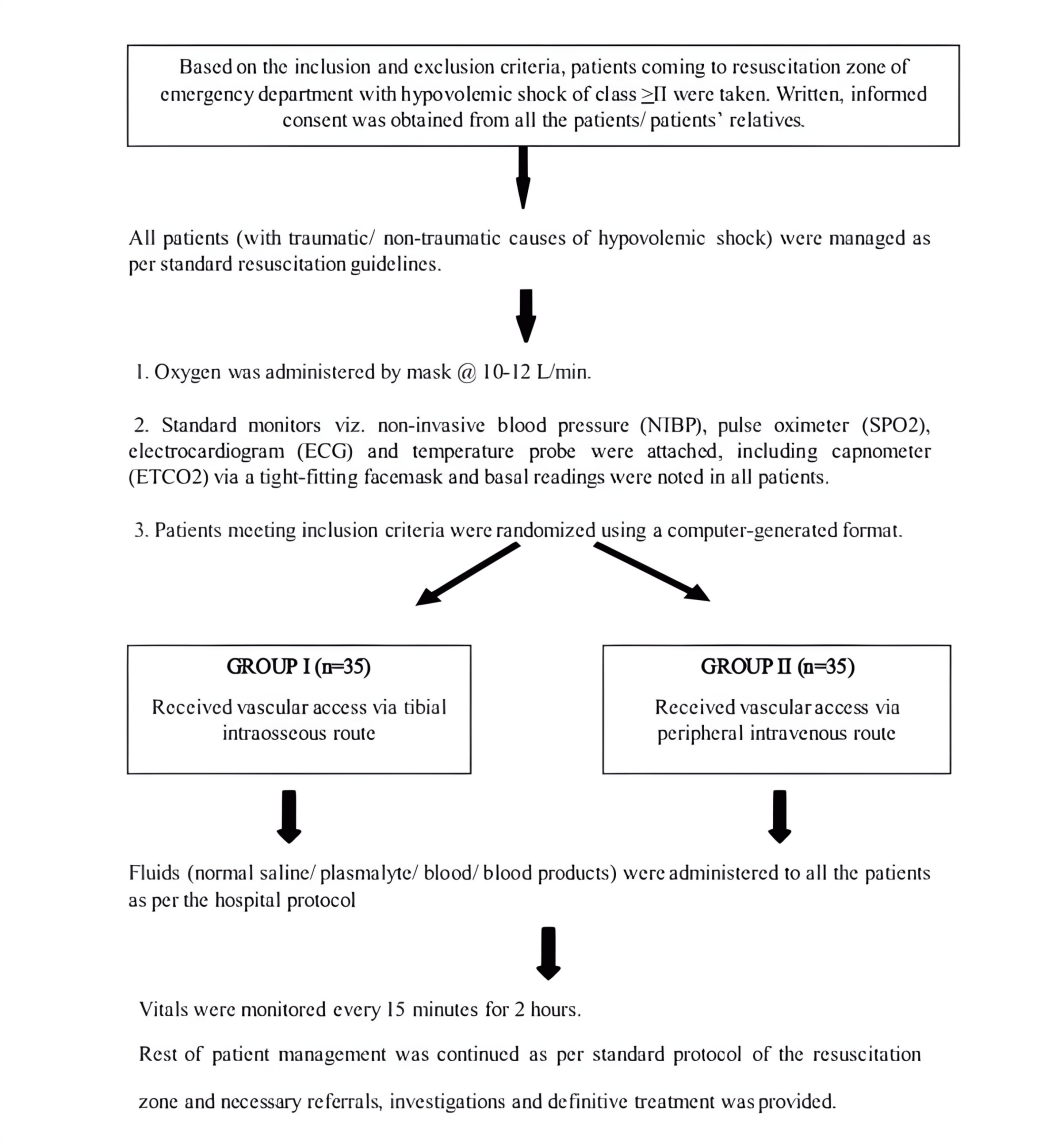
Patient inclusion, allocation and intervention

All 70 patients eligible for inclusion in this study had their first attempt at vascular access at one of the two locations: proximal tibial intraosseous or peripheral intravenous. This was done using block randomization with a sealed envelope system. Ten blocks of sealed opaque envelopes were prepared, with each block containing seven envelopes for Group I and seven for Group II. Once the envelope was opened, the patient received vascular access based on the group assigned (figure). The proximal tibial insertion site was located 1-2 cm below and medial to tibial tuberosity. Peripheral intravenous cannula placement was done at any accessible peripheral vein. If three attempts at peripheral venous cannulation were unsuccessful, vascular access was secured using the intraosseous route in Group II. The intraosseous access was secured only by doctors trained in IO needle insertion using the EZ-IO® device which was available in the emergency department of the hospital (Figure 1) [10].

Outcome measures

The primary outcome measure was to compare the first-attempt success rate and time taken to successfully establish an initial vascular access via proximal tibial intraosseous route/ peripheral intravenous route. The time taken to establish vascular access in group I included cleaning and draping of the site of insertion, and visible flow of the fluids through the IO access was taken as the end point. In group II, the time taken to establish vascular access included cleaning the site of cannulation with alcohol and free flow of fluids through the IV line was taken as the end point. Secondary outcome measure included ease of establishment of vascular access via proximal tibial intraosseous route/ peripheral intravenous route using a five-point Likert scale. The grades of the Likert scale in our study were as shown in Table 2.

**Table 2:** Grades of Likert scale

Points	Interpretation
1	Very Difficult
2	Difficult
3	Neutral
4	Easy
5	Very Easy

Statistical analysis

The presentation of the categorical variables was done in the form of numbers and percentage(%). Quantitative data were presented as mean ± SD and as median with 25th and 75th percentile (interquartile range). Quantitative variables were analyzed using an Independent t-test. Qualitative variables were analyzed using the Chi-Square test. If any cell had an expected value of less than five, then Fisher’s exact test was used. Data entry was done in the Microsoft Excel spreadsheet, and final analysis was done using Statistical Package for Social Sciences (SPSS) software, version 25.0 (IBM Corp., Armonk, NY). For statistical significance, a p-value of less than 0.05 was considered statistically significant.

## Results

Population characteristics

The characteristics of the study population are provided in Table 3.

**Table 3:** Polulation characteristics overall and by assigned route of vascular access The data has been represented as N, % and Mean+/-SD

Population Characteristics	Group I (n=35)	Group II (n=35)	Total Population
Gender			
Female	10 (28.57%)	9 (25.71%)	19 (27.14%)
Male	25 (71.43%)	26 (74.29%)	51 (72.86%)
Age (years) Mean +/- SD	35.2 +/- 14.31	38.54 +/- 13.32	36.87 +/- 13.83
Cause of Hypovolemic Shock			
Taumatic	33 (94.29%)	32 (91.43%)	65 (92.86%)
Non-Traumatic	2 (5.71%)	3 (8.57%)	5 (7.14%)

Main results

The proportion of patients in whom vascular access was successful on the 1st attempt was significantly higher in group I as compared to group II. The number of patients who required a single attempt was 35 (100%) in Group I vs 16 (45.71%) in Group II, respectively (p value <0.0001). Since the peripheral IV route was not established in two cases in three attempts in Group II, they were considered as failures, and vascular access was established using the IO route. Mean ± SD of time taken to establish vascular access (seconds) in group I (77.86 ± 2.59) and group II (77.52 ± 30.05) had no significant difference between them (p value=0.948). Median (25th-75th percentile) of time taken to establish vascular access (seconds) in group I was 77 (76-80) and in group II was 83 (49-99) with no significant difference between them (p value=0.830) (Table 4).

**Table 4:** Primary outcome measures Data has been represented as N, %, Mean+/-SD and Median. Fischer's exact test, Independent-t test and the Mann-Whitney U were the statistical tests used. A p-value <0.05 is considered significant.

Parameter	Group I ( IO Route, n=35)	Group II ( Peripheral IV Route, n=35)	Test used	p value
Number of Attempts	35	60		
Successful	35 (100%)	33 (94.2%)		
First attempt success rate	35 (100%)	16 (45.71%)	Fischer’s exact test	<0.0001
Mean time +/- SD (seconds)	77.86 +/- 2.59	77.52 +/- 30.05	Independent t-test ( t-value = 0.067)	0.948
Median time (25th-75th percentile)	77(76-80)	83(49-99)	Mann-whitney U test (Mann-whitney U = 595.000)	0.830

The difference in time taken to establish vascular access between group I and group II (in terms of Mean and Median) was statistically insignificant but clinically significant. The proportion of patients with a Likert score 'Very easy' was significantly higher in group I as compared to group II (Very easy: 35 (100%) vs 10 (28.57%), respectively). The proportion of patients with Likert scores Very difficult, Difficult, Neutral, and Easy was significantly higher in group II as compared to group I (Very difficult: two (5.71%) vs 0% respectively, Difficult: four (11.43%) vs 0% respectively, Neutral: 13 (37.14%) vs 0% respectively, Easy: Six (17.14%) vs 0%, respectively) (p value <0.0001) (Table 5).

**Table 5:** Comparison of ease of establishing vascular access (Likert score) The data has been represented as N and %. Mann-whitney U is the statistical test used. A p-value <0.05 is considered significant.

Likert Score	Group I (n=35)	Group II (n=35)	Total	Mann-Whitney Test	p value
Very difficult	0 (0%)	2 (5.71%)	2 (2.86%)	Mann-Whitney U=175.000	<0.0001
Difficult	0 (0%)	4 (11.43%)	4 (5.71%)		
Neutral	0 (0%)	13 (37.14%)	13 (18.57%)		
Easy	0 (0%)	6 (17.14%)	6 (8.57%)		
Very easy	35 (100%)	10 (28.57%)	45 (64.29%)		
Total	35 (100%)	35 (100%)	70 (100%)		

## Discussion

In this interventional study, we found that the first-attempt success rate of IO access was significantly higher than that of peripheral intravenous access, and the mean procedure time was also significantly reduced. Therefore, IO access is a promising route that helps in rapid initial fluid resuscitation in patients with hypovolemic shock and thus can prove to be life-saving in such patients. In our study, the establishment of intraosseous (IO) access was considered successful when the IO needle was securely placed in the bone marrow cavity with visible fluid flow through the IO line, without extravasation into the surrounding tissue. Peripheral intravenous (IV) access was considered successful when the cannula was securely placed in the peripheral vein, with visible blood back-flow, normal fluid flow, and no evidence of tissue infiltration. Our results showed that the first-attempt success rate for IO access (Group I) was significantly higher than that for peripheral IV access (Group II) (100% vs. 45.71%, respectively; p<0.0001).

Our findings align with those of Torres et al., who conducted a prospective study on 107 critically ill patients in a pre-hospital setting [18]. When peripheral IV access failed, IO access was attempted at multiple sites (proximal tibia, distal tibia, radius, and humerus) using the EZ-IO® device, achieving a 100% first-attempt success rate, which was consistent with our results [18]. Similarly, Reades et al. compared tibial IO, humeral IO, and peripheral IV routes in adult out-of-hospital cardiac arrest patients [12]. The first attempt success rates were 91% for tibial IO, 51% for humeral IO, and 43% for peripheral IV access, demonstrating that the tibial IO route is significantly superior to the other methods (p < 0.001) [12]. Differences in these percentages may be attributed to paramedics performing IO insertions in a pre-hospital environment, compared to our study, which was conducted by resident doctors in a controlled emergency department setting.

Paoli et al. further reported a 95.51% first attempt success rate with the EZ-IO® device in a pre-hospital emergency setting involving both adult and pediatric patients [19]. Although their success rate was slightly lower than ours, this may be due to the multiple IO insertion sites used in their study, compared to the exclusive use of the proximal tibia in ours. Additionally, their insertions were performed by a mix of medical personnel and nurses, while in our study, all insertions were performed by resident doctors.

Reinhardt et al. reported a 95.4% first-attempt success rate in adults and 89.5% in children using the EZ-IO® device, with the majority of insertions (97.7%) at the proximal tibia [20]. The lower success rate in their study may be attributed to the incorrect needle size selection, particularly in obese patients or those with large tissue masses at the insertion site. Similarly, Ngo et al. reported a 91.4% first-attempt success rate for IO access in patients where peripheral IV cannulation took more than two attempts or 90 seconds [13]. Helm et al. retrospectively analyzed IO access in 348 patients during helicopter emergency medical services (HEMS) missions and found an 85.9% first attempt success rate, which is lower than our findings, potentially due to the inclusion of pediatric patients and the use of multiple insertion sites (proximal tibia, distal tibia, proximal humerus) [21]. Kim et al. reported a higher first-attempt success rate with IO than IV (90.4% vs 75.5%) [6]. Taken together, these studies demonstrate that the IO route consistently achieves a higher first attempt success rate compared to peripheral IV access, supporting our conclusion that IO access is a superior method for establishing vascular access in critically ill or emergency patients.

In our study, the time taken to establish IO access included the entire process from cleaning and draping the site to injecting local anesthetic, and ending when visible fluid flow through the IO line was confirmed. For peripheral IV access, the time was measured from cleaning the site to the point of free fluid flow through the IV line, accounting for up to three attempts when necessary. The mean time for IO access was 77.86 ± 2.59 seconds, while for IV access, it was 77.52 ± 30.05 seconds. The median time for IO access was 77 seconds (76-80), compared to 83 seconds (49-99) for IV access. Although the differences were not statistically significant (p = 0.948 for mean, p = 0.830 for median), the IO route demonstrated more consistent timing, with less variability compared to the IV route, which is clinically important in emergency settings.

Reades et al. reported similar findings in their study, where the median time for tibial IO access was 4.6 minutes, compared to 7.0 minutes for humeral IO access and 5.8 minutes for peripheral IV access [12]. The longer times in their study may be due to paramedics performing the procedures in the field, compared to our controlled emergency department environment. Lamhaut et al. demonstrated that in training simulations, IO access was significantly faster than peripheral IV (50 ± 9 seconds vs. 70 ± 30 seconds, p < 0.001), though this study used manikins and included both physicians and nurses, unlike our live patient study [22]. Banerjee et al. found that in pediatric patients, IO access was significantly faster than peripheral IV access (67 ± 7 seconds vs. 129 ± 13 seconds, p < 0.001), though the use of spinal and hypodermic needles for IO insertions in their study differs from the EZ-IO® device used in ours [23].

Furthermore, in our study, the ease of establishing vascular access was evaluated using a five-point Likert scale. Group I (IO access) consistently received a score of 5 ("very easy"), while Group II (IV access) showed greater variability. In Group II, 28.57% of insertions were rated as "very easy," but a significant proportion were rated as "neutral," "difficult" or "very difficult." These findings indicate that IO access was perceived as significantly easier to perform compared to IV access (p < 0.0001).

In a retrospective analysis by Helm et al., 93% of IO insertions were rated as "easy" or "good," while 6.6% were deemed "difficult [21]. This variability is expected given the subjective nature of the Likert scale. Ngo et al. also reported that 88.6% of physicians found IO insertion easier than peripheral IV cannulation, a result consistent with our findings [13]. Horton et al. reported that the powered IO device was easy to use 71% of the time [24]. Shina et al. [25] reported that the ease of establishing IO access using the EZ-IO® device was 4 on the 5-point Likert scale, similar to Feldman et al. [26], suggesting that IO vascular access is easy to establish using a powered device, as demonstrated by our study.

Thus, our study demonstrates that intraosseous access, particularly when using the EZ-IO® device, offers a significantly higher first-attempt success rate compared to peripheral intravenous access. Although the time to establish vascular access was statistically similar between the two methods, the greater consistency and ease of IO access highlight its clinical advantage in emergency and critical care settings. These findings reinforce the growing body of evidence supporting the use of IO access as a reliable and efficient method for rapid vascular access, particularly when IV access is challenging or time-consuming.

While our study provides important insights into the use of intraosseous (IO) access in emergency settings, several limitations should be acknowledged: (a) single-center study: the study was conducted in a single tertiary care center, which may limit the generalizability of the results to other settings, particularly pre-hospital environments or smaller hospitals where staff experience and available resources may differ, (b) sample size: although our study included a sufficient number of participants to demonstrate statistically significant differences in the first attempt success rate, the relatively small sample size may not fully capture the variability of patient conditions, staff expertise or environmental factors that could influence vascular access outcomes, (c) operator experience: the IO and IV insertions were performed by resident doctors, all of whom had prior training in the use of the EZ-IO® device. This may limit the applicability of the findings to less experienced personnel, such as paramedics or junior medical staff, who may not achieve the same level of success, (d) limited scope of patient population: our study population included only adult patients in an emergency department setting. The findings may not be directly applicable to paediatric patients or to patients in out-of-hospital scenarios, where establishing vascular access is often more challenging, (e) single insertion site: all IO insertions were performed at the proximal tibia, which is considered the most common and accessible site. The success rate and ease of insertion may differ at other IO access sites, such as the humerus or distal tibia, which were not assessed in this study, (f) potential for bias in ease of insertion: The evaluation of ease of insertion was based on subjective assessments using a Likert scale, which introduces the potential for observer bias. Different operators may have varying thresholds for what they consider “easy” or “difficult” and this could affect the consistency of the ratings, (g) exclusion of failed IV access attempts beyond three: in our study, peripheral IV access was attempted up to three times before being considered unsuccessful. This limitation may have led to underreporting of cases where IV access could have been established on a fourth or fifth attempt, thus slightly skewing the comparison between IO and IV success rates, (h) exclusion of certain complications: while the study focused on the success rate and time to establish access, it did not comprehensively evaluate all potential complications associated with both methods. Factors such as infection, extravasation, and long-term outcomes of IO access were not studied, limiting the overall risk assessment of each method, (i) short follow-up period: our study only evaluated the immediate success and ease of establishing vascular access. Long-term outcomes related to the effectiveness and safety of IO access, such as post-procedural complications and the duration of patency, were not assessed.

While the findings of our study strongly support the use of IO access in emergency situations, particularly when peripheral IV access is challenging, these limitations should be taken into account. Further research, including larger, multi-center studies with a broader patient population, is recommended to validate our results and explore the applicability of IO access in diverse clinical settings.

## Conclusions

This study provides compelling evidence supporting the use of tibial intraosseous (IO) access as a superior alternative to peripheral intravenous (IV) access for fluid resuscitation in adult patients presenting with hypovolemic shock in emergency settings. The IO route, facilitated by the EZ-IO® device, demonstrated a 100% first-attempt success rate, markedly outperforming the peripheral IV route, which achieved a success rate of only 45.71%. This statistically significant difference highlights the reliability and efficiency of the IO method, especially in critical scenarios where rapid vascular access is essential and peripheral veins are often collapsed or inaccessible.

Although the average time required to establish vascular access via the IO and IV routes did not show a statistically significant difference, the IO approach displayed a more consistent and predictable performance with a narrower range of completion times. This consistency is particularly valuable in high-pressure environments such as emergency departments, where even minimal delays in initiating fluid resuscitation can adversely affect patient outcomes. Furthermore, the IO route was overwhelmingly rated as “very easy” to establish by clinical staff, in stark contrast to the IV route, which received a broader and less favorable distribution of ease-of-use ratings on the Likert scale.

These findings reinforce the clinical utility of intraosseous access in emergent resuscitation, particularly in patients with severe hypovolemia. The simplicity and speed of the EZ-IO® system contribute to its practical advantage, enabling healthcare providers to secure reliable vascular access quickly and with minimal training. In light of these results, future research should aim to explore the effectiveness of IO access in broader clinical contexts, including multi-center studies across diverse patient populations, prehospital environments, and low-resource settings, in order to validate and expand the clinical application of this life-saving intervention.. Comparative studies evaluating different IO insertion sites, device types, and long-term safety outcomes will be crucial in refining best practice guidelines. Furthermore, integrating IO access training into emergency medicine curricula and standard resuscitation protocols may enhance preparedness among healthcare providers and ensure timely, effective care for critically ill patients.
